# Jenner Monument

**Published:** 1857-09

**Authors:** 


*Jenner Monument.*—A bronze statue of Jenner has recently been successfully cast, and is to be placed at London in an appropriate site not yet designated. This monument is a tribute from all nations, subscriptions having been collected from different countries, and, for the most part, from members of the medical profession. We are gratified to see that the sum contributed by the medical profession of America exceeds that from any other country. It is nearly double the amount collected in the country honored by the birth of Jenner. America has transmitted 340 pounds; England 196 pounds—the latter including 25 pounds from Prince Albert. Russia comes next to America in the amount of contribution, the sum being 241 pounds.

If we were to estimate the claim to the tribute of a monument by the services rendered to mankind, so long a time should not have elapsed since the death of Jenner and this act of acknowledgment. It is a significant fact that a monument is reared at this late day, by contributions, chiefly from medical men, (the only class whose interests were affected unfavorably by the discovery of vaccination,) of all countries. But a fact still more significant is this—scarcely one in ten of the intelligent members of society are cognizant even of the name of the discoverer of vaccination! Military heroes who have sacrificed hecatombs of human lives to objects of personal ambition or state aggrandisement are remembered with sentiments of admiration, and their memories honored by monuments. Scarcely a town in Great Britain is without its statue of Wellington. But the far greater man and the benefactor of his race who, after twenty years of investigation, achieves a discovery which has nearly eradicated the most loathesome and fatal diseases, receives a pitiful acknowledgement of his services during life, and as a tardy

---

*Annuaire des Science Médicales, 1856, p. 145.

tribute to his memory a single class of society—a class whose intelligence and benevolence, God be praised! exceeds their wealth—unite to erect a bronze statute, costing a little more than a thousand pounds! Here is food for moralizing which we leave with our readers. *

